# Implementation of After-Hours Nurse Line in an Academic Pediatric Endocrinology Practice

**DOI:** 10.1155/2023/2550101

**Published:** 2023-09-11

**Authors:** Abha Choudhary, Soumya Adhikari, Perrin C. White

**Affiliations:** Division of Pediatric Endocrinology, University of Texas Southwestern Medical Center, 5323 Harry Hines Boulevard, Dallas, TX 75390-9063, USA

## Abstract

**Background:**

After-hours triage of pediatric patients by trained nurses improves consistency of triage decisions, access, and quality of care, and decreases burden on physicians on-call. There is a lack of published experience with this approach in the pediatric diabetes population.

**Methods:**

An after-hours call service was established in September 2019 in our large urban pediatric teaching hospital. Barton Schmitt guidelines, which are widely accepted as the standard for telephone triage care, were modified to include institution specific diabetes management protocols. We analyzed demographics, reasons for call, clinical presentation to the emergency room, and clinical disposition of the callers.

**Results:**

The after-hours call service handled 70% of calls without physician involvement. There were no patients triaged to home care who subsequently required an emergency room visit or hospitalization. Patients who called the after-hours nurse line prior to coming to the emergency room were less sick and were discharged more often from the emergency room. Spanish-speaking parents utilized the service less than English speakers. There were no disparities in utilization based on the insurance status or race.

**Conclusions:**

The after-hours service accurately triaged calls and reduced physician burden. Patients of all races and insurance statuses utilized the after-hours service equally well. Language was a barrier in the utilization.

## 1. Introduction

After-hours telephone triage by trained nurses using clinical algorithms has become an important part of general pediatric health care in the United States [1, 2] and internationally. In the United Kingdom, National Health Service Direct, a state-funded nationwide call center, was launched in 1998. Around 40% of the calls were related to pediatric care [3]. The Kids Kare Line was established in 1993 in Australia [4, 5], and primary pediatric call centers have also been operating in Switzerland since 1996 [6] and in the Netherlands [7]. Nurse triage increases the consistency of triage decisions, improves call documentation and access to care, and decreases unnecessary emergency room visits [8–10]. It is efficient in terms of the response time to calls to the service, and effective in the way care is delivered [4]. It is well received by patients, parents and primary physicians [4, 11]. Studies have reported patient satisfaction rates of 94%–99% for hospital based pediatric nurse triage systems [1]. After-hours services often refer to the on-call physician for second level triage, allowing a physician from the patient's practice to make the final decision regarding the necessity of an emergency room referral [1, 2, 11].

Optimal management of childhood and adolescent diabetes benefits from access to a 24-hr support line. However, if staffed by on-call physicians, repeated interruptions to sleep can lead to physician fatigue and burn out, while limitations in the pediatric endocrinology work force in recent years have led to more frequent call coverage needs [12]. To the best of our knowledge, utilization and outcomes of an after-hours nurse call service in a pediatric endocrinology practice have not been previously reported. In 2019, we established such a service at our hospital to decrease the call burden on both attending physicians and fellows and also improve the consistency of triage decisions. The present study describes the effects of establishing this service as determined by utilization, call disposition, and associations with eventual emergency department outcomes, when applicable.

## 2. Materials and Methods

### 2.1. Setting

Our practice is based at Children's Medical Center Dallas, a large urban pediatric teaching hospital licensed for 487 beds, with a 72-bed satellite facility 22 mi (35 km) north. It includes 17 attending physicians, 5 advanced nurse practitioners, and 6 fellows. We follow ∼2,500 patients with diabetes, who had 6,325 office visits in 2021. We admitted 371 patients in 2021 for new onset diabetes.

### 2.2. Institution of an After-Hours Call Service

Calls to our practice during the workday (0800–1630, Monday through Friday) are taken by a team of nurses who are Certified Diabetes Care and Education Specialists (CDCES). Prior to establishing the service, our practice utilized advanced practice nurses (APN) for after-hours calls from 1630 to midnight Monday–Thursday with attending physician backup. The on-call physician took calls from midnight to 0800 and all day on weekends; this was either an attending physician or a pediatric endocrinology fellow with attending backup.

An after-hours call service was first launched in December 2013 in our Children's hospital to support the community physicians. We partnered with this team, which then consisted of ∼40 triage nurses employed by our hospital system, to establish the after-hours service in September 2019. The team currently supports Endocrine, ENT, Concussion, Food Allergy, Neuro-Headache, Epilepsy, Pulmonology Asthma, Cystic Fibrosis, Cardiology, Complex Care, Foster Care, Tele schools, and over 100 community pediatric practices. They utilize registered nurses with pediatric clinical experience and specialized training in telephone triage. For endocrinology, eligible calls are transferred to the after-hours team from 1630 to 0800 on weekdays and from 0800 to 0800 on weekends and holidays. These include all parent calls related to diabetes and prescription refills. The on-call physician is available as backup for second level triage or if there is a management dilemma. Nondiabetes endocrinology related questions, questions from families of patients with diabetes <2 years old, those on diluted insulin and insulin pump failure questions are handled directly by the on-call physician. Calls from our emergency room and physicians at our hospital are directly transferred to the on-call physician. Calls from outside hospitals requesting transfers are routed by the hospital's access center to the on-call attending physician regardless of whether a fellow was on call.

We developed a phone system to support triage of endocrinology patients. Barton Schmitt telephone triage protocols [13], which are widely accepted as the standard for telephone triage care [8, 9], were modified to include pediatric and institution-specific diabetes management protocols. The lead physician edited the existing Barton Schmitt's high-blood glucose and low-blood glucose protocols to include institution specific management (the hyperglycemia protocol is included in the Appendix). She also created additional protocols for ketone/sick day management, pump management, missed insulin doses, and guidelines for prescription refills. The lead physician presented the protocols to the team and sent them around to the physicians of the division, APNs, and CDCES's for review and feedback. After six rounds of edits, the protocols were finalized. The lead physician, APN team lead and CDCES team lead met with the after-hours nurses to discuss the protocols and provided education to the triage nurses. The protocols were made available to after-hours nurses via a decision support tool embedded in Epic, our electronic medical record system. The nurses used specialized questions that guided them through a sequence of questions and answers which prompted a recommended triage disposition. Documentation was completed by the nurse and routed to the patient's physician. Disposition options included: (a) go to emergency room now, (b) call specialist now, (c) home care, and (d) nonurgent MD calls. The parent was permitted to override the recommended triage disposition and management and insist on speaking with the on-call physician if they requested. When non-English-speaking (usually Spanish speaking) parents called, they were put on hold and a translator connected to the call.

All the calls were recorded for quality improvement. After-hour telephone call notes in the Epic electronic health record were automatically routed to the attending endocrinologist of record for each patient. Additionally, all call notes were evaluated by the lead physician monthly for appropriate management and documentation, and to look for protocol deviations. During the study period, the lead physician audited 10 randomly selected recorded calls to understand the interaction with the patient and evaluate management. The on-call physicians also reported concerns to the lead physician regarding management, who provided timely feedback to the triage nurse leadership team. This review was utilized to modify protocols and address educational needs. Data on utilization of the after-hours service, reasons for and dispositions of calls were shared at the monthly division meeting. Feedback was shared with the triage nursing leadership team in meetings that were initially semimonthly and eventually spaced out to monthly intervals. An annual refresher using Powerpoint slides was presented by one CDCES and the APN lead. This session was recorded and was reviewed, in person or virtually, by all triage nurses.

### 2.3. Analysis

The University of Texas Southwestern Institutional Review Board approved this study. Data on utilization of the after-hours call service were collected prospectively from October 1, 2019, to September 30, 2022, and included after-hours call volumes, reasons for call and call dispositions. Clinical data were obtained using system analysis program (Walldorf, Germany) analytics to interrogate a Clarity database derived from our institutional Epic (Madison, WI) clinical data repository [14]. We examined clinical presentations to the emergency room including initial glucose and pH and analyzed the likelihood of discharge from the emergency room versus hospital admission, based on utilization of the after-hours services.

To minimize the possibility of misclassification of patients as having Type 2 versus Type 1 diabetes, all patients are routinely classified for diabetes type in our clinical database by the treating endocrinologist using discrete patient tags (termed “smart data elements” in Epic). Both inpatient admissions and observations, but not emergency room visits, were counted as hospital admissions if associated with ICD10 codes of E10.10, E10.11, and E10.65. Demographic data included age, duration of diabetes, the family's preferred language, insurance and race. Insurance status was coded as “commercial” or “non-commercial.” Race and ethnicity were recoded as a single variable with values of “White or Caucasian,” “Hispanic,” “Black or African American,” or “Other,” in our region, almost all Hispanics are of Mexican origin. In addition to data on utilization of the call line, we collected the same demographic data on all patients with at least one clinic visit during the study period, and also data on hemoglobin A1c and technology pump and continuous glucose monitor use, selecting data current at the most recent visit. We noted whether each such patient had utilized the call line at least once during the study period.

Call handle time was extracted monthly from the Cisco CCE phone system reports. Patient satisfaction data were collected from May 1, 2022, to September 30, 2022. A text message was sent to each patient the day after the call with a link to a survey. The survey was sent in Spanish if the family was designated as Spanish speaking in Epic.

Protocol adherence was evaluated depending on whether the triage nurse opened the relevant protocol in the Epic-based decision support tool.

Statistical analysis was conducted using SAS 9.4. The contribution of explanatory factors to the odds of utilizing the call line was evaluated by the multivariate logistic regression. The contribution of explanatory factors to pH and glucose at arrival in the emergency room was assessed with generalized linear models. Other associations were assessed with *χ*2 tests or Mann–Whitney tests, and the differences between means were assessed by *t*-tests.

## 3. Results

After-hours call data were reviewed from October 1, 2019, to September 30, 2022. There were 3,230 total calls during this period to the after-hours service from 1,106 unique callers, 951 of which were calls to the on-call physician through the after-hours line, and 77 calls through the direct physician line (31% of the total calls, an average of <1 per night). The median (IQR) monthly call volume was 88. Although there were fewer calls during the first 2 months of the COVID pandemic (April and May of 2020), the volume of calls increased over time. The mean ± SD endocrinology call handle time (time spent with the patient) was 10.07 ± 5.42 min. Protocol adherence for the nurse triage team (usage of endocrine specific protocols) was 67% at the start of the study period and improved to 79% during January–September 2022. The overall adherence was 76% during the study period.

Most patients utilizing the service could be managed at home with advice from the nurses (1,811/3,230, 56%); this category was referred to as “home care.” Of 2,025 calls referred to home care or to nonemergent physician follow-up (213, 7%, none had ED visits within 1 month of the call. Thus, the nurse line had an under-referral rate of 0%.

**Figure 1:**
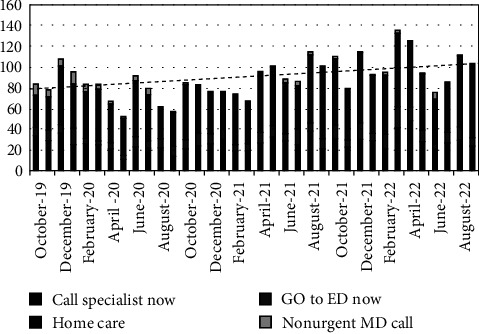
Call volume and disposition. Call volume is on the *y*-axis, and each bar represents 1 month. Each indicated category of disposition is shaded differently. A dotted trend line was calculated by the linear regression.

**Figure 2:**
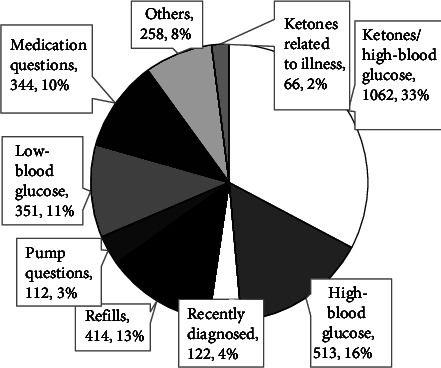
Reason for call. Slices have distinct shading for clarity only. The label for each slice includes the percent of total calls with that stated reason.

The endocrinologist was contacted for 951 calls (29%), referred to as “call specialist now.” Ten percent of the patients (350/3,230) who called the after-hours line were sent to the emergency room (Figure 1). The most common reasons for calling were ketosis with hyperglycemia followed by the hyperglycemia alone and prescription refills (Figure 2). Families that called at least once had children that were younger, had a shorter duration of diabetes, and spoke English versus Spanish in comparison to families that never utilized the service. There were no differences based on the insurance status or race/ethnicity. Patients with higher hemoglobin A1C and patients who used pumps and/or continuous glucose monitors were more likely to use the after-hours line (Tables 1 and 2).

Patients who had previously called the after-hours line were more likely to be discharged from emergency room visits instead of being admitted, but only 8.4% of diabetes patients visiting the emergency room for hyperglycemia or ketosis called first (Table 3). Patients who called the after-hours line first had higher pH and lower glucose at initial presentation, even when age and race/ethnicity were included as explanatory factors (Table 4). We wanted to make sure that there was not a “shift effect” given that the after-hours service was available only during certain hours. The after-hours line was indeed utilized most often by patients arriving in the emergency room between 1,600 and 2,400, but there was no effect of arrival time on discharge probability (Table 5). Similarly, patients who called our CDCES line during the day before visiting the emergency room were also more likely to be discharged home.

Starting in May 2022, the hospital began sending surveys to users of the help line via the MyChart patient portal, with Spanish-language surveys sent to identified Spanish-speaking families. There were only 16 responses from Endocrinology patients (out of 473 calls made) in May–September 2022, of which only 1 was in Spanish. All responders would use the service again, and 15 rated the service 5/5. These findings were similar to survey responses from all users of the nurse line in the same period, including those from general pediatrics and other subspecialties: few survey responders were Spanish speakers (27/818, 3.3%). The nurse line service was viewed very favorably among survey responders with no differences based on language. 756/780 (96.9%) of English speakers would use the service again versus 26/27 Spanish speakers (96.3%). On a 5-point scale, 687/757 (90.8%) of English responders rated the service as 5 versus 18/20 (90%) of Spanish responders.

## 4. Discussion

The after-hours nurse triage service was able to handle 70% of calls from families that otherwise would have gone to on-call physicians. Patients who called the after-hours line before coming to the emergency room were less sick (higher pH and lower glucose) and were more likely to be discharged home. Patients who called the CDCES line during the workday (0800–1630, Monday through Friday) were also more likely to be discharged if they were sent to the emergency room. We cannot infer causality from our data but given that <10% of patients called first, encouraging earlier and more consistent utilization of the nurse line seems like a low-cost intervention that might reduce hospital admissions for diabetic ketoacidosis. This idea is consistent with our previous observation that hemoglobin A1c and prior hospitalization are independent risk factors for the future admissions, suggesting that effective sick day management may be a learned parental skill that is somewhat independent of maintaining good glycemic control.

The change in pediatric practices from an on-call physician model to a model in which nurses triage calls using protocols was initially driven by the desire to reduce the after-hours workload for pediatricians [9], but there may be additional benefits [15]. A study of call centers in children's hospitals demonstrated that physicians spent 3–5 min on each call, whereas call center nurses spent an average of 11.2 min [2]. This improved patient satisfaction and adherence to treatment plans. It also reduced costs [16, 17], improved patient safety [18–20], and reduced the workload of general practitioners [15, 21, 22]. In these prior studies, the rate of under-referral resulting in subsequent hospitalization was 0.3%; we had no cases of under-referral in our series. There was a significant reduction in emergency room visits—from 22%–25% to 11%—when those cases judged urgent by call center nurses underwent secondary triage by the physicians [19]. The lower figure is similar to our experience.

There are racial and ethnic disparities at the national level in treatment and outcomes of children with Type 1 diabetes [23, 24]. Therefore, it is noteworthy that we found no biases in utilizing the nurse line based on the insurance status or race, which are factors that influence hospital admission rates [14]. However, Spanish-speaking families were less likely to utilize the call line, even though translator services were available. This is consistent with differences in telemedicine access seen during the COVID-19 pandemic, with Spanish-speaking patients being less likely to use video visits [25]. Patients with limited English proficiency may utilize the health care resources less because of dissatisfaction with the health care they have received in the past. Other studies have shown that patients who communicated through an interpreter or who did not have an interpreter when they thought one was necessary were less satisfied with the patient-provider relationship [26]. We had an insufficient number of Spanish-survey responders to gauge satisfaction with our service.

It is difficult to interpret associations between the nurse line utilization and glycemic control or related behaviors. Families that utilized CGM and pumps—devices associated with improved glycemic control—were more likely to call the nurse line, yet patients utilizing the nurse line tended to have a higher rather than lower HbA1c.

## 5. Conclusions

Institution of an after-hours nurse line for calls from parents of children with diabetes reduced burdens on physicians. Patients referred by the nurse line to the emergency room were less sick and more likely to be discharged home but represented only a small fraction of emergency visits made by diabetes patients in our practice.

## Figures and Tables

**Table 1:** Demographics of after-hours service utilization.

Effect		Utilized call service?					*p*
		No		Yes			
		Mean	SD	Mean	SD		
Age, y		15.05	3.50	13.27	4.23		<0.001
Diabetes duration, y		4.76	4.11	4.23	3.52		<0.001
HbA1c, %		8.47	2.30	8.58	2.06		NS
		*N* (%)		*N* (%)		Total	
Language							
English		2031 (83%)		1015 (92%)		3046	<0.001
Spanish		398 (16%)		83 (8%)		481	
Other		19 (1%)		8 (1%)		27	
Insurance							
Noncommercial		1108 (45%)		610 (55%)		1718	<0.001
Commercial		1340 (55%)		496 (45%)		1836	
Race–ethnicity							
White		875 (36%)		512 (46%)		1387	<0.001
Hispanic		844 (34%)		263 (24%)		1107	
Black		552 (23%)		243 (22%)		795	
Other		177 (7%)		88 (8%)		265	
Pump?							
No		1988 (81%)		774 (70%)		2762	<0.001
Yes		460 (19%)		332 (30%)		792	
CGM?							
No		1398 (57%)		396 (36%)		1794	<0.001
Yes		1050 (42%)		710 (64%)		1760	

**Table 2:** Odds of after-hours service utilization.

Parameter		*B*	SE	Wald	*p*	Odds ratio	95% CI	
Intercept		−0.269	0.223	1.46	NS			
Age	Per year	−0.088	0.011	65.69	<0.001	0.92	0.90	0.94
Diabetes duration	Per year	−0.035	0.012	8.68	0.003	0.97	0.94	0.99
HbA1c	Per %	0.087	0.019	21.32	<0.001	1.09	1.05	1.13
Pump use?	Yes versus No	0.151	0.050	9.09	0.003	1.35	1.65	1.11
CGM use?	Yes versus No	0.316	0.045	48.79	<0.001	1.88	2.25	1.58
Insurance	Commercial versus noncommercial	0.043	0.045	0.93	NS	1.09	0.92	1.30
Language	Spanish versus English	−0.234	0.078	8.99	0.003	0.63	0.46	0.85
Race–ethnicity	Black versus White	0.023	0.078	0.09	NS	0.92	0.74	1.14
	Hispanic versus White	−0.061	0.082	0.54	NS	0.85	0.67	1.06
	Other	−0.069	0.110	0.40	NS	0.84	0.63	1.13

*Note:* Factors in the “*B*” column are log-odds ratios; each corresponding odds ratio = e^B^. CI = confidence interval.

**Table 3:** Likelihood of discharge from emergency department based on after-hours service or certified diabetes educator (CDCES) utilization.

			After-hours service utilization ^*∗*^		
			Yes	No	Total
Discharged?					
Yes		*N*	82	502	584
		%	43.2	24.2	
No		*N*	108	1,570	1,678
		%	56.8	75.8	
Total		*N*	190	2,072	2,262
					
			CDCES utilization^a^		
			Yes	No	Total
Discharged?					
Yes		*N*	33	237	270
		%	42.9	28.1	
No		*N*	44	606	650
		%	57.1	71.9	
Total		*N*	77	843	920

*Note:* ^*∗*^*p* < 0.001; ^a^*p* < 0.01. CDCES data are for 1 year.

**Table 4:** Glucose and pH at emergency department arrival.

Parameter	Estimate	Standard error	*p*
pH at emergency department arrival			
Intercept	7.348	0.013	<0.001
Age, y	−0.001	0.001	0.042
Race–ethnicity			
White	0		
Black	−0.034	0.008	<0.001
Hispanic	−0.006	0.007	
Other	−0.024	0.015	
After-hours call? No	0		
Yes	0.028	0.011	0.009
Glucose at emergency department arrival			
Intercept	373.1	17.3	<0.001
Age, y	−3.2	0.9	0.001
Race–ethnicity		.	
White	0.0		
Black	21.1	10.0	0.035
Hispanic	−14.9	9.6	
Other	7.6	19.6	
After-hours call? No	0.0		
Yes	−66.3	13.9	<0.001

**Table 5:** After-hours service utilization and emergency department discharges by shift.

			Shift^a^			Total
			Day	Evening	Overnight	
After-hours service utilization by emergency department visitors ^*∗*^						
After-hours utilization						
Yes		*N*	60	104	26	190
		%	5.8	11.5	8.2	
No		*N*	977	803	292	2,072
		%	94.21	88.53	91.82	
Emergency department discharges^+^						
Discharged?						
Yes	*N*		256	240	88	584
	%		24.7	26.5	27.7	
No	*N*		781	667	230	1,678
	%		75.3	73.5	72.3	
Total	*N*		1,037	907	318	2,262
Total after-hours service calls						
Weekdays	*N*		41	799	187	1,141
Weekends	*N*		322	302	49	724
Total	*N*		363	1,101	236	1,865

*Note:* ^a^Shifts are Day, 0801–1600; Evening, 1601–0000; Overnight, 0001–800;  ^*∗*^*p* < 0.001; ^+^*p*=NS.

## Data Availability

The data will be made available upon request by contacting the corresponding author.

## Additional Points

*Limitations*. To our knowledge, the use of a protocol-driven after-hours nurse line to help manage pediatric diabetes patients has not been reported previously. This is a single center study, potentially limiting its applicability in other settings, although there is substantial evidence that such nurse lines are useful in general pediatrics. We could only analyze 1 year of CDCES data because prior to 12/2021, CDCES calls were not internally documented in a way that allowed us to reliably identify the sick calls; however, the data for a year are numerically sufficient for comparison with the after-hours line. We had an insufficient number of survey responses to identify barriers to more widespread utilization of the nurse line by our patient population. Finally, the data do not allow us to show causally that utilization of the nurse line reduces the likelihood of hospitalization.

## Supplementary Materials

Supplementary Materials An example of the protocol is added to this section. The modified high-blood sugar protocol is outlined in detail.
