# Supplementary material for: Implementation of After-Hours Nurse Line in an Academic Pediatric Endocrinology Practice
Source: Pediatr Diabetes. 2023 Sep 11;2023:2550101. doi: 10.1155/2023/2550101 (PMC12017214; doi:10.1155/2023/2550101)
Supplement: Supplementary Materials — An example of the protocol is added to this section. The modified high-blood sugar protocol is outlined in detail. [file 2550101.f1.docx]

Appendix: Example of protocol

Each triage assessment question has care advice listed as numbers to guide the triage nurses to use the correct protocols.

**Diabetes - High Blood Sugar**

Pediatric After-Hours Version - Standard - 2017

**DEFINITION**

* Child with known diabetes mellitus

* Has symptoms related to high blood sugar (hyperglycemia)

* Has a high blood sugar defined as a blood glucose above 200 mg/dl (11 mmol/l)

* Has questions regarding high blood sugar

* Suspected new-onset diabetes can also be triaged here

**INITIAL ASSESSMENT QUESTIONS**

1.OTHER SYMPTOMS: "Does your child have any symptoms?" (e.g., fever, vomiting, excessive

thirst, frequent urination, increased breathing rate or fruity odor to the breath)

2.CHILD'S APPEARANCE: "How sick is your child acting?" " What is he doing right now?" If

asleep, ask: "How was he acting before he went to sleep?" "Can you wake him up?"

3. VOMITING Is your child vomiting?

4. BLOOD GLUCOSE: "What is your child's blood glucose level?" What are your child’s targets?

5.URINE OR BLOOD KETONES: "Do you check your child's urine or blood for ketones?" If yes, ask: "What does the test show now?"

*Urine ketones reported as trace, small, moderate, large.

*Blood ketone readings:

- Below 0.5 mmol/l = none to trace ketones
- 0.6-0.9 mmol/l = small ketones
- 1.0-1.5= moderate ketones
- Above 1.5 mmol/l = large ketones

6. USUAL RANGE: "What is your child's usual glucose level?" (e.g., usual fasting morning

value, usual evening value)2. ONSET: "When did you last check the blood glucose?"

7. TYPE 1 or 2: "Do you know what type of diabetes your child has?" (e.g., Type 1, Type 2,

doesn't know).

8. INSULIN: "Does your child take insulin?" If yes, ask: "What type of insulin(s) does your child

take? What is the mode of delivery? (syringe, pen, pump)

9. Does your child use an insulin pump? (Total daily dose located on pump).

If yes, ask:

- Which brand of the pump do they use?
- When was the last time the pump insertion site was changed?
- Any issues with the pump or site?
- What is the Total daily dose of insulin they use?

10. Does your child use insulin injections? If yes, ask the following:

- What is the dose of the long-acting insulin? (Long-acting insulins are Lantus (Glargine) / Levemir / Basaglar / Tresiba / Toujeo) FYI – Levemir is often twice daily.
- What is the insulin dose/ratio at mealtime?
- What is their sliding scale insulin regimen?

11. What is the Total daily dose of injected insulin (if they use syringes or pen)?

- 12. DIABETES PILLS: "Does your child take any pills for his diabetes?" If yes, ask: "What type of

pill(s) does your child take and what is the usual dose? When was the last dose? Has your child

missed any doses recently?"

- Author's note: IAQ's are intended for training purposes and not meant to be required on every call.

**TRIAGE ASSESSMENT QUESTIONS**

**Call EMS 911 Now**

Unconscious or difficult to awaken.

*R/O: hypoglycemia, DKA*

*CA: 50, 14, 16, 1*

Acting confused (e.g., disoriented, slurred speech)

*R/O: hypoglycemia, DKA*

*CA: 50, 14, 13, 16, 1*

Very weak (e.g., can't stand)

*R/O: hypoglycemia, DKA, shock*

*CA: 50, 14, 13, 16, 1*

Sounds like a life-threatening emergency to the triager

*CA: 50, 14, 13, 16, 1*

**See More Appropriate Guideline**

Fever and age > 3 months (12 weeks or older)

*Go to Guideline: Fever - 3 Months or Older (Pediatric)*

**Go to ED Now**

[1] Vomiting AND [2] signs of dehydration (e.g., very dry mouth, no tears, etc)

NOTE: Urine output will be high in diabetics with hyperglycemia

*Reason: may need IV hydration, possible DKA*

CA: 51, 12, 27, 1, *C1*

[1] Blood glucose > 240 mg/dl (13 mmol/l) AND [2] rapid breathing

*R*/O: DKA

CA: 51, 12, 27, 1, C1

Blood glucose > 500 mg/dl (27.5 mmol/l) AND [2] vomiting AND/OR [3] rapid breathing

CA: 51, 27, 1, C1

[1] Blood glucose > 240 mg/dl (13 mmol/l) AND [2] urine ketones moderate-large (or more than 1+), AND (3) no improvement with Ketone and Hydration protocol for four hours.

*R/O: DKA*

*CA: 51, 27, 1, C1*

[1] Blood glucose > 200 mg/dl (13 mmol/l) AND [2] type 2 diabetes (not insulin-dependent) AND [3] vomiting.

*Reason: hyperglycemia*

*CA: 51, 25, 27, 1*

[1] Blood glucose > 240 mg/dl (13 mmol/l) AND [2] vomiting AND [3] not tolerating oral fluids (per hydration protocol).

*R/O: DKA*

*CA: 51, 27, 1, C1*

[1] Vomiting > 2 hours AND [2] not tolerating oral fluids (per hydration protocol).

*R/O: DKA, dehydration*

*CA: 51, 27, 1, C1*

New-onset diabetes suspected by triager (e.g., excessive drinking, frequent urination, weight loss)

*R/O: DKA*

*CA: 51, 1*

Child sounds very sick or weak to the triager

*Reason: severe acute illness or serious complication suspected*

*CA: 51, 27, 13, 14, 16, 1*

**Call Specialist Now**

[1] Known diabetic with symptoms of high blood sugar (e.g., excessive thirst, frequent urination,

weak) AND [2] not able to test blood glucose (no meter or glucose “HI”)

*CA: 59, 6, 1, C1*

[1] Caller has URGENT medication or insulin pump question AND [2] triager unable to answer

Question

*CA: 59, 1,8*

**Call Specialist within 24 Hours**

[1] Caller has NONURGENT medication or insulin pump question AND [2] triager unable to answer

Question

*Reason: obtain Specialist input regarding insulin dosing*

*CA: 60,8 1*

**Home Care**

[1] Blood glucose >400 AND [2] negative ketones and [3] newly diagnosed (less than 6 months).

*CA: 58, 30, 31, 24, 25, 8, 6, 1*

[1] Blood glucose >400 AND [2] negative ketones and [3] known diabetic (more than 6 months).

*CA: 58, 32, 24, 25, 8, 6, 1*

[1] Blood glucose > 240 mg/dl (13 mmol/l) AND [2] urine ketones present (small-moderate-large or more than 1+)

*Reason: Increased risk for DKA*

CA: *24, 1, 6,* ***8, C1***

Urine ketones present and small, moderate to large

*Reason: Usual treatment for ketones includes extra insulin*

*CA: 1, 6,* ***8, C1***

[1] Urine ketones moderate to large and [2] blood glucose >240 mg/dl and [3] USES A PUMP FOR INSULIN DELIVERY.

*Reason: Usual treatment for ketones includes extra insulin*

*CA: 1, 8, 33, C1*

[1] Vomiting < 4 hours AND [2] blood glucose < 240 mg/dl (13 mmol/l) AND [3] tolerating oral fluids (per hydration protocol).

*CA: 15, 1,* ***8, C1***

[1] Child is sick AND [2] doesn't have previous instructions from PCP or specialist for insulin adjustment (sliding scale)

*CA: 19, 1,* ***C1***

Missed long acting (Lantus/Levemir/Basaglar) dose (Typically long acting insulin is given at bedtime)

*Reason: hyperglycemia*

*CA: 58, 5, 4, C2, 2, 3, 10, 23, 8, 7, 1, C1*

[1] Blood glucose > 240 mg/dl (13 mmol/l) AND [2] type 1 diabetes (insulin-dependent)

*Reason: hyperglycemia*

*CA: 58, 5, ~~6,~~ 4, 2, 3, 10, 23, 8, 1, C1*

[1] Blood glucose > 200 mg/dl (13 mmol/l) AND [2] type 2 diabetes (not insulin-dependent)

*Reason: hyperglycemia*

*CA: 5, 6, 11, 2, 3, 23, 8, 1, 10, 54*

Blood glucose 70 - 239 mg/dl (4 -13 mmol/l)

*CA: 58, 5, 22, 3, 2, 10, 23, 1*

Sick day rules for Type 1 Diabetes, questions about

*CA: 58, 19, 23, 8, 1*

Sick day rules for Type 2 Diabetes, questions about

*CA: 58, , 19, 23, 8, 1*

**CARE ADVICE (CA) -**

1.] CARE ADVICE given per Diabetes - High Blood Sugar (Pediatric) guideline.

2.] KEEP A DAILY RECORD:

* For Children on Insulin: Measure your child's blood glucose before each meal, before going

to bed and other times your child's doctor recommends. Record the results and show them to

your doctor at your next office visit.

* For Children not on Insulin: Measure your child's blood glucose before breakfast and going

to bed. Record the results and show them to your doctor at your next office visit.

3.] DAILY BLOOD GLUCOSE GOALS


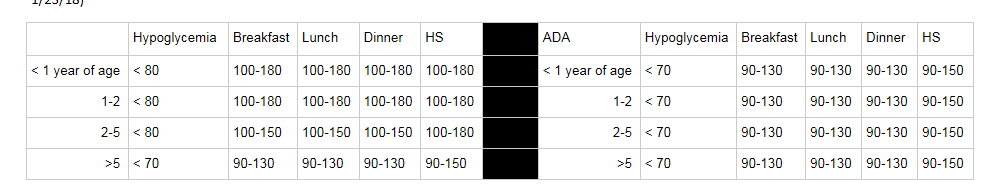


4.] CONTINUE INSULIN - USE A SLIDING SCALE IF ALREADY PRESCRIBED:

* Continue your child's insulin, as prescribed by your child's doctor.

* Sliding Scale Insulin: If your doctor has given you instructions to take extra fast-acting

insulin when your child's blood sugar is high, give your child the insulin dose your doctor has

recommended.

5.] HIGH BLOOD SUGAR (HYPERGLYCEMIA) - DEFINITION:

* Definition - Fasting blood glucose over 140 mg/dL (7.5 mmol/l) or random blood glucose over

200 mg/dL (11 mmol/l).

* Symptoms of mild hyperglycemia - increased thirst, frequent urination, fatigue, blurred

vision.

* Symptoms of severe hyperglycemia - confusion and coma.

* Contributing factors: not taking medications as directed, not following recommended meal plan

6.] HIGH BLOOD SUGAR TREATMENT:

* Drink at least one ounce of sugar-free fluids (maximum 16 ounces) per year of age per hour for the next 4 hours. (Reason: adequate hydration will help reduce hyperglycemia).

* Generally, your teen should try to drink 6-8 cups of water each day.

* Younger children can drink less.

8.] CALL BACK IF:

* Urine ketones become moderate- large or continue to be moderate to large after intervention

* Vomiting occurs

* Rapid breathing occurs

* Fruity odor to breath

* No change with ketone protocol

* Other questions or concerns

*Your child becomes worse

10.] KETONE TEST KIT:

* All diabetics who require insulin therapy should keep a testing kit for urine or blood ketones

in their home.

* You can buy urine ketone dipsticks at your local pharmacy.

* Check urine or blood for ketones whenever your child is ill OR your child's blood glucose is above

~~300~~ 250 mg/dL (16.5 mmol/l).

* Read the ketone level at the correct time. Waiting too long may give a high reading. (follow brand instructions)

11.] CONTINUE MEDICATIONS:

* Continue giving your child diabetes pills.

12.] TRANSPORTATION OPTIONS:

* If immediate transportation is not available via car or taxi, then the caller should be

instructed to call EMS-911.

13.] FIRST AID ADVICE - GLUCOSE IF ABLE TO SWALLOW AND GLUCOSE less than

70 mg/dL (4 mmol/l) or UNKNOWN (pending EMS arrival):

* Give some sugar by mouth NOW.

*Each of the following is equivalent to 10-15 gms of

glucose (Choose only one):

* Glucose tablets: 3 or 4, glucose gel or ~~cake icing~~

* Fruit juice: 1/2 cup or 4 ounces (120 ml)

* Soft drinks (not diet): 5-6 ounces (150 ml)

* 5 to 6 hard candies that you can chew quickly if age appropriate (6yr or older)

* For conscious patients, unable to self-treat as above: Glucose gel or cake decorating gel: 1 tube (3 teaspoons or 15 ml). Place between cheek and gum and massage. Elevate head and encourage patient to swallow. Treat with gel until alert.

* Wait 15 minutes and then check your blood sugar again

- If it is still low, repeat above (eat/drink something with 15 gm of sugar)
- Eat a meal/snack when glucose is > 70 mg/dl. If next meal or snack is greater than 1 hr treat with 15 gm of long acting carbs i.e., (peanut butter and crackers, granola bar, sandwiches.)

14.] FIRST AID ADVICE - GLUCAGON IF UNABLE TO SWALLOW AND GLUCOSE less

than 70 mg/dL (4 mmol/l) or UNKNOWN (pending EMS arrival) AND SEVERE SYMPTOMS

(seizure or unconscious):

* WHEN patient has glucagon for hypoglycemic emergencies, encourage the caller to give

the glucagon now.

* NOTE TO TRIAGER: Glucagon comes in 1 mg powder vial that needs to reconstituted

before giving. Tell the caller to follow the kit's instructions.

* Inject it SC or IM into the upper outer thigh.

* Dosage over 44 pounds or 20 kg: 1 mg = 1 cc or 100 units in insulin syringe

* Dosage under 44 pounds or 20 kg: 0.5 mg = 1/2 cc or 50 units in insulin syringe

15.] FLUIDS FOR VOMITING:

Follow ketone protocol.

16.] NOTE TO TRIAGER - FIRST AID FOR UNKNOWN BLOOD GLUCOSE:

* Hypoglycemia is an emergency.

* Direct the caller to give GLUCAGON or GLUCOSE even if the blood glucose is unknown.

* If it turns out the blood glucose is high, the hospital can treat this easily.

19.] SICK DAY RULES - DIET CHANGES:

* Appetite OK, minimal nausea - Continue your child's normal diabetic meal plan. Avoid spicy

or greasy foods.

* Appetite fair, moderate nausea - Give your child a bland diet. Try small amounts of food 6-8

times a day. Offer food or liquids every 1-2 hours.

* Follow hydration guidelines for poor appetite or severe nausea

* Advance diet as your child improves.

20.] REGULAR DIET AND MORE FLUIDS:

* Keep your child on a regular diet but give extra fluids (mainly water).

* Avoid fluids with sugar (e.g., soda). (Advice for suspected new diabetics.)

22.] GENERAL DIABETES ADVICE:

* Physician - See your child's physician regularly.

* Testing - Test your child's blood glucose. Follow your PCP's advice regarding how often.

* Record-keeping - Keep a daily record of the results of the tests.

* Medications - Give your child diabetes medications as prescribed.

* Eat healthy - Work with your child's doctor or a dietician to develop a healthy meal plan.

* Exercise - Staying physically active is important.

23.] INTERNET RESOURCES:

* Reliable educational information is available from:

* The federally funded National Diabetes Education Program. 1-800-438-5383. Web site:

http://ndep.nih.gov

* The American Diabetes Association. 1-800-diabetes. Web site: http://www.diabetes.org

24.] RECHECK BLOOD SUGAR:

* If you have not done so already, recheck your child's blood sugar to make certain that it is

really that high.

25.] DRINK EXTRA FLUIDS:

* Encourage your child to drink lots of water.

27.] BRING MEDS:

* Be sure to bring a list of your child's meds and insulin pump (if used) with you.

29.] FEVER MEDICINE AND TREATMENT:

* For fever above 102 F (39 C) or child uncomfortable, give acetaminophen every 4 hours OR

ibuprofen every 6 hours (See Dosage table).

* FOR ALL FEVERS: Give cool fluids in unlimited amounts (Exception: less than 6 months

old.) Dress in 1 layer of light-weight clothing and sleep with 1 light blanket. (Avoid bundling.)

Reason: overheated infants can't undress themselves. For fevers 100-102 F (37.8 to 39 C),

this is the only treatment needed. Fever medicines are unnecessary.

*Acetaminophen can interfere with blood glucose readings on continuous glucose monitor (CGM) except for G6.

30.] Follow ½ sliding scale insulin (SSI) and recheck glucose in 2 hours and check for ketones when possible. Call back in 2 hours if blood glucose is above >400 mg/dl or below 70 mg/dl or has ketones.

31.] Newly diagnosed patients (less than 6 months) who do not normally correct overnight: trend the numbers and report to the diabetes team when the office opens.

32.] Follow sliding scale insulin (SSI) and recheck glucose in 2 hours and check for ketones when possible. Call back in 2 hours if blood glucose is above >400 mg/dl or below 70 mg/dl or has ketones.

33.] Increase insulin pump temporary basal rate to 120% for 2 hours (increase of 20%) in addition to giving a bolus dose via subcutaneous injections and following the ketone protocol.

- Recheck blood glucose and ketones in 2 hours.
- Return to basal rate if none, trace, or small ketones after two hours.
- Consider repeating temporary basal rate of 120% for another 2 hours if ketones still moderate or large in 2 hours
- Call back if ketones remain moderate to large four hours after initial insulin correction dose.

50.] CALL EMS 911 NOW: Your child needs immediate medical attention. You need to

hang up and call 911 (or an ambulance). (Triager Discretion: I'll call you back in a few minutes

to be sure you were able to reach them.)

51.] GO TO ED NOW: Your child needs to be seen in the Emergency Department

immediately. Go to the ER at ___________ Hospital. Leave now. Drive carefully.

52.] GO TO ED NOW (OR PCP TRIAGE):

* IF NO PCP TRIAGE: Your child needs to be seen within the next hour. Go to the ER/UCC

at _____________ Hospital. Leave as soon as you can.

* IF PCP TRIAGE REQUIRED: Your child may need to be seen. Your doctor will want to talk

with you to decide what's best. I'll page him now. If you haven't heard from the on-call doctor

within 30 minutes, or your child becomes worse, go directly to the ER/UCC) at ___________

Hospital.

53.] SEE PHYSICIAN WITHIN 4 HOURS (or PCP triage):

* IF OFFICE WILL BE OPEN: Your child needs to be seen within the next 3 or 4 hours. Call

your doctor's office as soon as it opens.

* IF OFFICE WILL BE CLOSED AND NO PCP TRIAGE: Your child needs to be seen within

the next 3 or 4 hours. A nearby Urgent Care Center is often a good source of care. Another

choice is to go to the ER. Go sooner if your child becomes worse.

* IF OFFICE WILL BE CLOSED AND PCP TRIAGE REQUIRED: Your child may need to be

seen. Your doctor will want to talk with you to decide what's best. I'll page him now. If you

haven't heard from the on-call doctor within 30 minutes, call again. (Note: If PCP can't be

reached, send to UCC or ER.)

54.] SEE PHYSICIAN WITHIN 24 HOURS:

* IF OFFICE WILL BE OPEN: Your child needs to be examined within the next 24 hours. Call

your child's doctor when the office opens, and make an appointment.

* IF OFFICE WILL BE CLOSED AND NO PCP TRIAGE: Your child needs to be examined

within the next 24 hours. An Urgent Care Center is often a good source of care if your doctor's

office is closed. Go to _________ .

* IF OFFICE WILL BE CLOSED AND PCP TRIAGE REQUIRED: Your child may need to be

seen within the next 24 hours. Your doctor will want to talk with you to decide what's best. I'll

page him now. (Exception: from 10 pm to 7 am. Since this isn't serious, we'll hold the page

until morning.)

* IF PATIENT HAS NO PCP: Refer patient to an Urgent Care Center or Retail clinic. Also, try

to help caller find a PCP (medical home) for their child.

58.] HOME CARE: You should be able to treat this at home.

59.] CALL SPECIALIST NOW: You need to discuss this with your child's doctor. I'll page him now. If you haven't heard from the on-call doctor within 30 minutes, call again.

60.] CALL SPECIALIST WITHIN 24 HOURS: You need to discuss this with your child's doctor within the next 24 hours.

* IF OFFICE WILL BE OPEN: Call the office when it opens tomorrow morning.

* IF OFFICE WILL BE CLOSED: I'll page him now. (Exception: from 9 pm to 9 am. Since this

isn't urgent, we'll hold the page until morning.)

C1 Follow Hydration and Ketone Protocol

C2 Based on the length of time since the missed dose, provide the following options:

- If < 6 hours from when the dose is due, give entire dose
- If between 6 to 14 hours = 2 options
  - Give half dose now and half dose at the usual time. Return to usual schedule the following day.
  - Can correct blood glucose every 2 hours using short-acting/ sliding scale insulin (SSI) till the usual time of the long-acting dose. Check urine ketones
- If > 14 hours- correct blood glucose every 2 hours using short-acting/ sliding scale insulin (SSI) until the usual time of the long-acting dose. Can also give 2-3 hours before the usual time
- If they have missed the long-acting insulin Tresiba- it can be taken at any time when they remember dose may be given immediately if the next dose is greater than 8 hr to next scheduled dose

**FIRST AID**

FIRST AID ADVICE FOR HYPOGLYCEMIA - GLUCOSE

* IF CAN SWALLOW AND GLUCOSE < 70 mg/dL (4 mmol/l) or UNKNOWN (pending EMS

arrival), give some sugar by mouth NOW.

*Each of the following is equivalent to 10-15 gms of

glucose (Choose only one):

* Glucose tablets: 3 or 4, glucose gel or * Fruit juice: 1/2 cup or 4 ounces (120 ml)

* Soft drinks (not diet): 5-6 ounces (150 ml)

* 5 to 6 hard candies that you can chew quickly if age appropriate (6yr or older)

* For conscious patients, unable to self-treat as above: Glucose gel: 1 tube (3 teaspoons or 15 ml). Place between cheek and gum and massage. Elevate head and encourage patient to swallow. Treat with gel until alert.

FIRST AID ADVICE FOR HYPOGLYCEMIA - GLUCAGON

* IF UNABLE TO SWALLOW AND GLUCOSE < 70 mg/dL (4 mmol/l) or UNKNOWN (pending

EMS arrival) AND SEVERE SYMPTOMS (seizure or unconscious):

* WHEN patient has glucagon for hypoglycemic emergencies, encourage the caller to give the

glucagon now.

* Glucagon comes in 1 mg powder vial that needs to reconstituted before giving. Follow the kit's

instructions.

* Inject it SC or IM into the upper outer thigh.

* Dosage > 44 pounds or 20 kg: 1 mg = 1 cc or 100 units in insulin syringe

* Dosage < 44 pounds or 20 kg: 0.5 mg = 1/2 cc or 50 units in insulin syringe

**BACKGROUND INFORMATION**

SYMPTOMS OF HIGH BLOOD SUGAR (HYPERGLYCEMIA)

* High blood sugar is defined as a blood glucose > 200 mg/dl (11 mmol/l)

* Mild hyperglycemia - usually no symptoms

* Moderate hyperglycemia - polydipsia, polyuria, fatigue, blurred vision

* Severe hyperglycemia - confusion and coma

* Diabetic ketoacidosis (DKA) - fruity odor on breath, sleepiness, vomiting, rapid breathing,

weakness, confusion and coma

CAUSES OF HIGH BLOOD SUGAR (HYPERGLYCEMIA)

* Noncompliance with taking insulin or other diabetes medicines. Omission of insulin is the most

common cause. Malfunction of insulin pumps also occurs.

* Noncompliance with diabetes diet

* Infections, bacterial or viral increase insulin requirements, puberty

* Combination of these factors

DIABETES MELLITUS

* Diabetes mellitus is an endocrine condition in which patients have elevated blood glucose levels

(hyperglycemia). Diabetes is the most common endocrine disorder of childhood.

* The classic symptoms of untreated or under-treated diabetes are: polydipsia (excessive thirst),

frequent urination (polyuria), increased appetite (polyphagia) and involuntary weight loss.

* Insulin is a hormone produced by the pancreas to help process food. Eating food makes the

blood glucose rise and insulin makes the blood glucose fall by allowing the glucose to enter the

body's cells where it can be used for fuel.

* There are two forms of diabetes: Type 1 and Type 2.

TYPE 1 DIABETES

* Other names - Insulin Dependent Diabetes (IDDM), Juvenile Onset Diabetes.

* Physiology - There is no production of insulin by the body.

* Ketosis-prone - Patients with Type 1 diabetes are ketosis-prone, which means that if they do not

receive enough daily insulin shots their bodies cannot utilize glucose. This results in breaking

down fats and producing ketones. The ketones spill into the urine and can be measured. Patients

with Type I diabetes are susceptible to developing Diabetic Keto-Acidosis (DKA), a life-threatening

condition.

* Treatment - Insulin therapy is always required. Insulin is given subcutaneously at least twice

daily. Patients striving for better control of their blood glucose will take insulin more often than

twice a day or may utilize an insulin pump. Recent research (Berhe 2006) has documented that

an insulin pump can be used safely even in preschoolers.

* Onset - 50 - 75% of those with type 1 diabetes have their onset in childhood or adolescence.

Approximately 10% of all individuals with diabetes have type 1.

TYPE 2 DIABETES

* Other names - Non-Insulin Dependent Diabetes (NIDDM), Adult-Onset Diabetes. (It's becoming

common in teenagers)

* Physiology - In Type 2 diabetes, there is decreased insulin production and decreased sensitivity

to insulin.

* Not ketosis-prone - These patients are not prone to ketosis. However, DKA is present at initial

diagnosis in 6 to 30%.

* Treatment - The initial and most important treatments are exercise and weight loss. When these

measures fail, there are pills (oral antidiabetic agents) that can be prescribed to help the body

either make more insulin or use the insulin more effectively. Occasional patients require insulin

therapy.

* Onset - It most commonly develops in over-weight teens or adults. Currently, Type 2 diabetes is

20% of diabetes presenting in teenagers and 10% of diabetes in children.

DIABETIC KETOACIDOSIS (DKA)

* Definition - Blood glucose > 250 mg/dl (12 mmol/L) with acidosis and ketosis (urine ketones

moderate to large) or blood ketones > 1.5 mg/dl.

* Symptoms of DKA - In addition to symptoms of hyperglycemia, fruity odor on breath,

sleepiness, vomiting, rapid/deep breathing, confusion and coma.

* Causes - non-compliance with using insulin in type 1 diabetes, infection.

FIVE GENERAL TYPES OF INSULIN

* Rapid acting [Lispro (Humalog), Aspart (Novolog), Glulisine (Apidra)] – peaks at 30-60 minutes,

lasts 3-4 hours

* Short acting (Regular) - peaks at 2-4 hours, lasts 4-6 hours

* Intermediate acting (NPH) - peaks at 4-8 hours, lasts 16-20 hours

* Long acting [Glargine (Lantus), Determir (Levemir), Toujeo] – “peakless”, increases over 2-5 hours; lasts

18-24 hours (Note:smaller doses of levemir may only last 12 hours)

* Pre-mixed (70/30, 75/25, 50/50) - peaks at 2-12 hours

MODES OF INSULIN DELIVERY

* SYRINGE: Insulin is drawn up manually from a vial into an insulin syringe. Syringes are not

reused.

* PEN: A pre-filled syringe that allows you to mark the units (dose) you need and that dose is

delivered. Needles are changed in between injections, but the syringe can be reused until empty.

* PUMP: Short acting insulin is delivered continuously subcutaneously via an external pump. Can

be used in children with type 1 or type 2 diabetes. Can have infusion-site reactions with this method.

CONVERTING GLUCOSE LEVELS - MG/DL AND MMOL/L

* In the United States, glucose is typically measured using the units MG/DL.

* Nearly every country in the world (including Canada) measures glucose levels using the units

MMOL/L.

* To convert mmol/l of glucose to mg/dl, multiply by 18.

* To convert mg/dl of glucose to mmol/l, divide by 18 or multiply by 0.055.
